# Reduced RNA expression of the *FMR1* gene in women with low (CGG_n<26_) repeats

**DOI:** 10.1371/journal.pone.0209309

**Published:** 2018-12-21

**Authors:** Qi Wang, David H. Barad, Sarah K. Darmon, Vitaly A. Kushnir, Yan-Guang Wu, Emanuela Lazzaroni-Tealdi, Lin Zhang, David F. Albertini, Norbert Gleicher

**Affiliations:** 1 The Center for Human Reproduction, New York, NY, United States of America; 2 The Foundation for Reproductive Medicine, New York, NY, United States of America; 3 Department of Obstetrics and Gynecology, Wake Forest University, Winston Salem, NC, United States of America; 4 Department of Molecular and Integrative Physiology, University of Kansas Hospital, Kansas City, KS, United States of America; 5 Stem Cell and Molecular Embryology Laboratory, the Rockefeller University, New York, NY, United States of America; 6 Department of Obstetrics and Gynecology, Vienna University School of Medicine, Vienna, Austria; VU Medisch Centrum, NETHERLANDS

## Abstract

*Low FMR1* variants (CGG_n<26_) have been associated with premature ovarian aging, female infertility and poor IVF treatment success. Until now, there is little published information concerning possible molecular mechanisms for this effect. We wished to examine whether relative expression of RNA and the *FMR1* gene’s fragile X mental retardation protein (FMRP) RNA isoforms differ in women with various *FMR1* sub-genotypes (*normal*, *low* CGG_n<26_ and/or *high* CGG_n≥34_). This prospective cohort study was conducted between 2014 and 2017 in a clinical research unit of the Center for Human Reproduction in New York City. The study involved a total of 98 study subjects, including 18 young oocyte donors and 80 older infertility patients undergoing routine in vitro fertilization (IVF) cycles. The main outcome measure was RNA expression in human luteinized granulosa cells of 5 groups of FMRP isoforms. The relative expression of FMR1 RNA in human luteinized granulosa cells was measured by real-time PCR and a possible association with CGG_n_ was explored. All 5 groups of FMRP RNA isoforms examined were found to be differentially expressed in human luteinized granulosa cells. The relative expression of four FMR1 RNA isoforms showed significant differences among 6 FMR1 sub-genotypes. Women with at least one *low* allele expressed significantly lower levels of all 5 sets of FRMP isoforms in comparison to the *non-low* group. While it would be of interest to see whether FMRP is also decreased in the low-group we recognize that in recent years it has been increasingly documented that information flow of genetics may be regulated by non-coding RNA, that is, without translation to a protein product. We, thus, conclude that various CGG expansions of FMR1 allele may lead to changes of RNA levels and ratios of distinct FMRP RNA isoforms, which could regulate the translation and/or cellular localization of FMRP, affect the expression of steroidogenic enzymes and hormonal receptors, or act in some other epigenetic process and therefore result in the ovarian dysfunction in infertility.

## Introduction

The most common cause of the fragile X syndrome (FXS) is the impaired expression of the Fragile X mental retardation 1 (*FMR1*) gene, resulting from the unstable expansion of a CGG repeat in its 5’ untranslated region. Based on the size of the expansion, individuals are classified as *normal* (5–54 CGG repeats), *premutation* (55–200 repeats), or *full mutation* (>200 repeats) [1, 2]. Lack of expression of *FMR1* gene, which encodes a RNA-binding protein, fragile X mental retardation protein (FMRP), is responsible for the mental retardation and the associated pleiotropic clinical phenotype.

The *FMR1* gene is composed of 17 exons and undergoes alternative splicing which affects the presence of exon 12, 14, 15 and 17, and results in more than 20 predicted protein isoforms [3–6]. FMRP Isoform 1, the full-length protein, contains two K-homology domains and an arginine–glycine–glycine (RGG) box which are responsible for RNA-binding, a nuclear localization signal at its N-terminus and a nuclear export signal at C-terminus, suggesting shuttling between the nucleus and cytoplasm, and post-translational modification sites through phosphorylation and methylation [5, 7–12]. The largest expansions found in patients (>230 repeats) are abnormally methylated, leading to the silencing of *FMR1* gene transcription [13, 14].

As a result of alternative splicing, various expression of FMRP isoforms has been reported in several species. Ashley *et al*. have demonstrated that 4 out of 12 isoforms were predominantly expressed in various tissues from human and mouse, and the relative expression of these isoforms differed between tissues [3, 4]. In mouse brain and cultured neuron cells, 6 isoforms were identified as predominant out of 12 isoforms measured, and their levels varied even among different brain tissues [15]. In rat ovary, it has been reported that the *FMR1* gene was expressed in granulosa, theca and germ cells in all stages of follicular development and its mRNA levels (total *FMR1*) decreased in pre-ovulatory follicles compared to preantral and antral follicles [6]. FMRP levels increase with follicular development, exhibiting at least 4 protein bands detected by Western blot [14]. However, the types of isoforms and their relative expression in the ovary are unclear.

The association of premutations of the *FMR1* gene with primary ovarian insufficiency has been widely reported [16–18]. Further defining the effect of the gene within normal CGG ranges (CGG_n<55_) into so-called “ovarian” mutation ranges, we previously demonstrated specific genotypes and sub-genotypes associated with distinct ovarian aging patterns (as defined by functional ovarian reserve assessed by anti-Müllerian hormone levels), autoimmunity, morphological embryo quality and infertility treatment outcomes [19–22]. A recent large study of FMR1 impact on IVF outcomes found lower AFC, lower AMH levels and that fewer oocytes were retrieved in the presence of fewer CGG repeats [23]. Thus, the *FMR1* mutation with most profound negative effects on female reproductive success was demonstrated to be the *low* mutation (CGG_n<26_) [19–21, 23].

How CGGn, however, may regulate FMR1 gene expression in the ovary, and thereby affects ovarian function and in vitro fertilization (IVF) outcomes, is not known. Whether expression and cellular localization of FMR1 isoforms correlate to the CGG repeats expansion still needs to be clarified.

The classical molecular model accepts that DNA is transcribed to messenger RNA and then translated to a protein. In recognition of this, it has been required that evidence be provided that a reduction of RNA corresponds to a reduction of the encoded protein in order to establish an observation as a significant biological effect. However, in recent years it has been increasingly documented that information flow of genetics may be regulated by non-coding RNA, that is, without translation to a protein product.[24, 25]

We, therefore, in this study aimed to investigate whether relative expression of FMR1 RNA (and its isoforms) differ in infertile women with various ranges of CGGn (defined as 6 sub-genotypes).

## Materials and methods

### Patient population and institutional review board

This study involved granulosa cells derived from adult women who had consented to undergo an IVF cycle. None of the tissue donors were from a vulnerable population. Since these granulosa cells were destined to be discarded the need for consent was waived by the Center for Human Reproduction Institutional Review Board that approved this study for expedited review and the subsequent data was analyzed anonymously.

Luteinized granulosa cells were obtained from 98 patients, during routine IVF treatments at the Center for Human Reproduction (CHR) in New York City. All patients studied underwent controlled ovarian hyper-stimulation and oocyte maturation by human chorionic gonadotropin (hCG) according to previously described standardized protocols [26, 27], followed by transvaginal ultrasound-guided oocyte retrieval, approximately 36 hours following human chorionic gonadotropin (hCG) administration.

### *FMR1* testing

*FMR1* testing is routinely performed on all patients in our practice as part of their initial work-up. CGG_n_ in the *FMR1* gene was assessed by commercial assays, with *FMR1* variants (genotypes and sub-genotypes) defined as described in prior publications [21, 22]. In brief, by defining a normal “ovarian” range of CGG_n = 26–34_, CGG counts below and above that range are considered abnormal. Subjects with both *FMR1* alleles in normal range are considered normal (*norm*); those with one allele outside normal range as heterozygous (*het*) and those with both alleles outside norm range as homozygous (*hom*). Sub-genotypes (*het-norm/high*, *het-norm/low*; *hom-high/high*, *hom-high/low*, *hom-low/low*) further define patient sub-groups, based on whether alleles are above (*high*) or below (*low*) normal range.

Based on the above sub-genotypes, the study population was divided into two study groups, *low* patients containing at least one *low* allele (*het-norm/low*, *hom-low/low* and *hom-high/low*) and *non-low* patients carrying no *low* alleles (*norm*, *het-norm/high* and *hom-high/high*).

### Granulosa cells Isolation

On the day of oocyte retrieval, follicular fluids with minimal blood contamination were collected. After isolation of oocytes, intact groups of granulosa cells were picked up from follicular fluid with a sterile glass pipette (Origio Inc, US) and washed twice in sterile D-PBS (LifeGlobal, USA) at room temperature to further minimize blood contamination. Granulosa cell pellets were collected after centrifugation (2,000 rpm, 5min) and kept at -80°C until RNA extraction.

### Protein sequence alignment and primer design

Protein sequences of various FMRP isoforms were obtained from the NCBI database (Iso1, Accession # NP_002015; Iso6, NP_001172004; Iso7, NP_001172005; Iso9, NP_001172011; Iso12, NP_001172010; IsoA, AHW56477; IsoB AHW56476; IsoC, AHW56478; IsoD, EAW61303; IsoE, EAW61297; IsoF, EAW61299; IsoG, EAW61300). BLAST (Basic Local Alignment Search Tool) is a search tool available from the NCBI that finds regions of similarity between biological sequences. The program compares nucleotide or protein sequences to sequence databases and calculates the statistical significance. Multiple protein sequence alignments among all isoforms were performed using BLASTP on the NCBI website [28] (http://blast.ncbi.nlm.nih.gov/Blast.cgi). Primers targeting middle and C-terminal regions of FMRP protein sequences were designed using the OligoAnalyzer 3.1 (Integrated DNA Technologies) and oligos were custom synthesized (Table 1).

**Table 1:** Sequences of primer sets used for real-time PCR.

Primer Sets		Sequence (5'-3')
FMRP	Forward	AAGTCCAGAGGGTGTTAGTGG
376	Reverse	ATGGTACCATACCCTGCCAA
FMRP	Forward	CCTGAACTATTTAAAGGAAGTAGACC
426	Reverse	GTGGTGGTCTAGAACTAGCT
FMRP	Forward	ACGCGGTCCTGGATATACTTC
491–1	Reverse	TCACTGAGTTCGTCTCTGTGG
FMRP	Forward	ACGCGGTCCTGGATATACTTC
491–2	Reverse	CCCTCTCTTCCTCTGTTGGAG
FMRP	Forward	GGAAGAACAACAGATGGATCCC
576	Reverse	CCTTCACTGGAGGTATTCTGTAATG
18S	Forward	AGTCCCTGCCCTTTGTACACA
	Reverse	GATCCGAGGGCCTCACTAAAC

### RNA extraction and real-time PCR

Total RNAs of granulosa cells were extracted using RNeasy mini Kit (Qiagen, USA), and 1 μg of total RNAs was converted to cDNA using reverse transcription enzyme (Invitrogen, US) according to the manufacturer’s instruction. relative quantification of the expression of the RNA transcripts of *FMR1* genes was analyzed by real-time PCR using the StepOne real-time PCR system (Applied Biosystems, US) normalized to 18S rRNA. Cycling conditions for PCR were 94°C for 5 min, then 40 cycles of 94°C for 20 s, 54–60°C (vary based on primers) for 20 s and 72°C for 30 s. Melting curves were routinely determined to ascertain that only the expected PCR products had been generated. Various *FMR1* isoforms were examined using 5 sets of primers targeting middle and C-terminal sequences. Data were analyzed by 2^-ΔΔCT^ method and normalized with 18S rRNA [29]. The final result is presented as the fold change of target gene expression relative to a reference sample, normalized to the reference gene.

### Statistics

The baseline characteristics of subjects in the study were analyzed by ANOVA (6 sub-genotypes) or t-test (*Non-low* vs. *low* group) and all values are presented as mean ± standard deviation. We also analyzed CGG repeats as a continuous variable using regression analysis against the relative expression of RNA transcripts among each of the five studied FMRP isoforms, controlling for possible confounders. The relative expression of RNA transcripts for the five studied FMRP isoforms was also analyzed by ANOVA analysis using the Tukey HSD post hoc test to control for multiple comparison (6 sub-genotypes) or t-test (*Non-low* vs. *low* group). A P-value of <0.05 was considered statistically significant. All statistical analyses were performed using SAS version 9.4 software.

## Results

### Patients

Between 2014–2017, a total of 98 subjects were enrolled in this study, including 14 young oocyte donors and 84 older infertility patients undergoing routine in vitro fertilization (IVF) cycles. The participants ranged in age from 21 to 47 years (average age 37.4 ± 6.9 years). These patients had an average length of infertility of 4.4 ± 2.1 years. Sixty (61.2%) were white, twenty (20.4%) were Asian, nine (9.2%) were Black, eight (8.2%) were Hispanic, and one (1%) was a Pacific Islander. Among the 84 infertility patients 71 (84.5%) had diminished ovarian reserve as a primary diagnosis. Of the remaining thirteen patients four had male factor, three had PCOS, one had endometriosis, one had tubal disease, one had IVF for gender selection and one for fertility preservation.

### Expression of isoforms of the *FMR1* gene in granulosa cells: *Norm FMR1* allele

**Fig 1:**
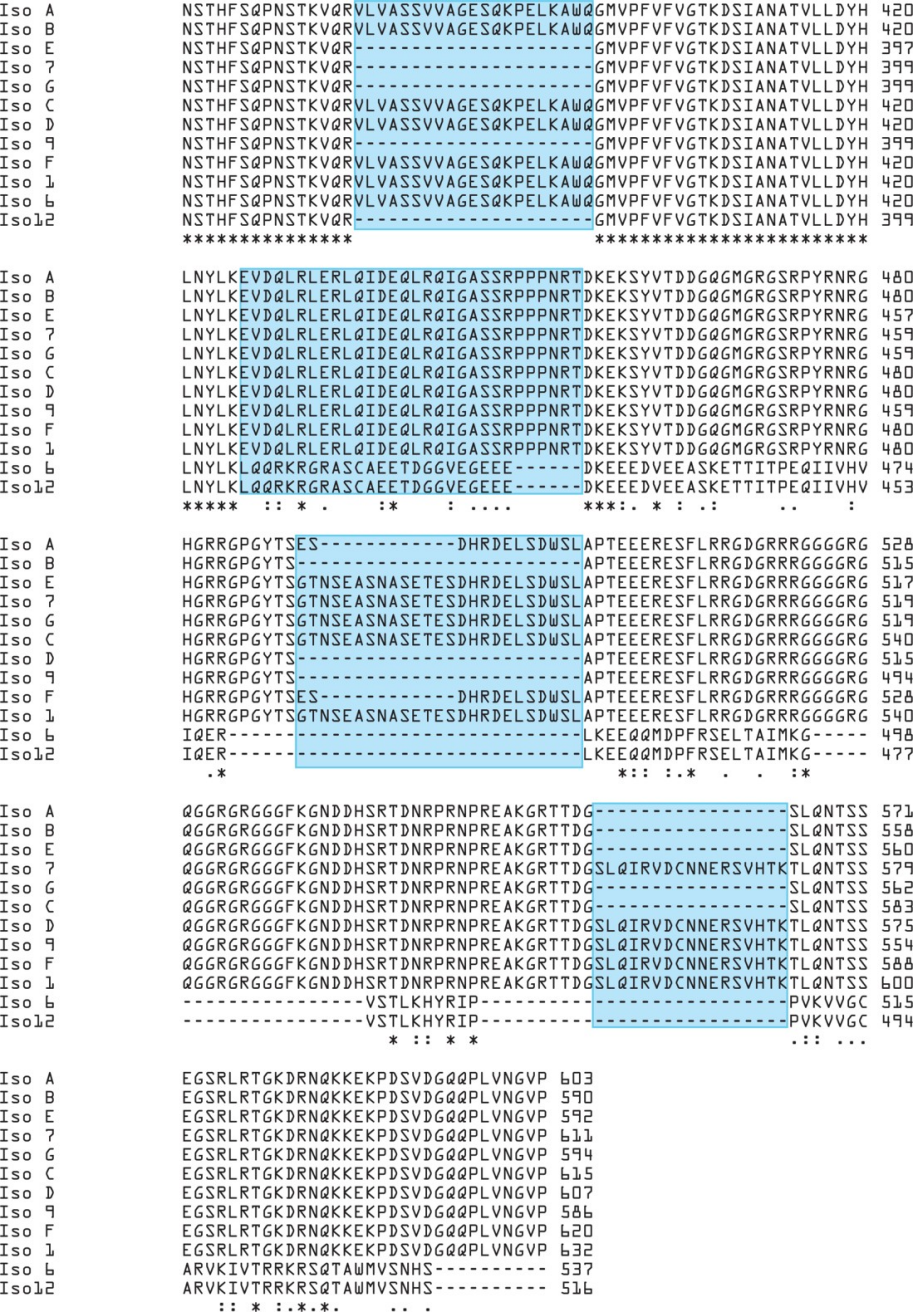
Sequence alignment of 12 known isoforms of human FMRP. Partial alignment result is shown to compare the differences. N-terminus sequences are identical among these isoforms. Box in blue indicates the region amplified by various sets of primers, asterisk represents identical residues among all isoforms, and dot/colon shows similarity of residues.

Multiple sequence alignment was performed based on the protein sequences of 12 isoforms of FMRP (**Fig 1**).

**Fig 2:**
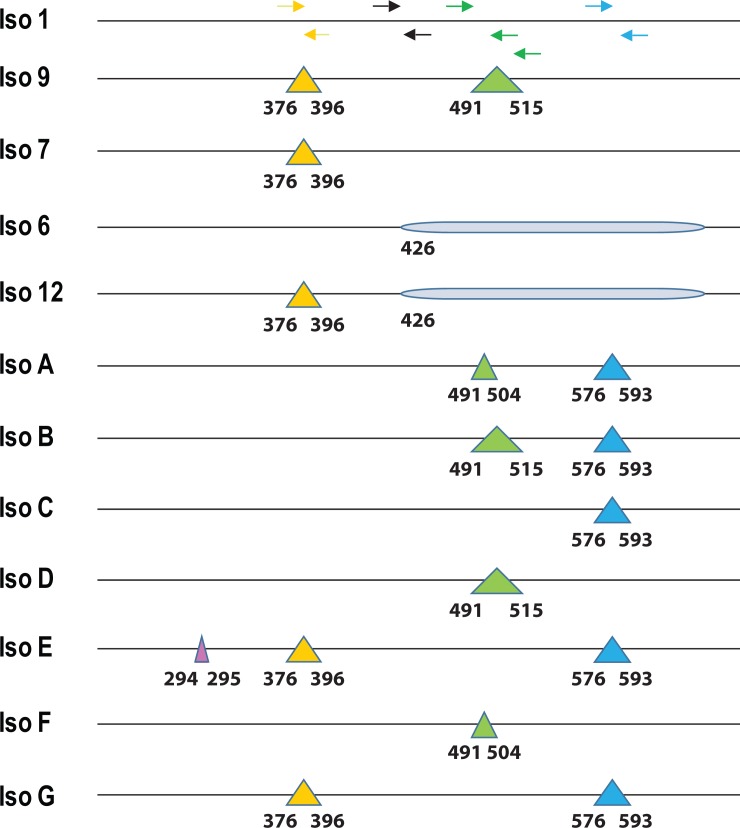
Schematic representation of the primer sets. Triangles in color indicate the deletion of amino acids at specific residue positions. Arrowheads show the position and direction of each primer. Region in shade suggests a frame shift due to alternative splicing in isoform 6 and 12.

Sequence variations among these isoforms were mainly located in the middle and C-terminus of FMRP. Five sets of primers were designed targeting the middle and C-terminus of FMRP protein sequences in order to examine the expression of these isoforms (**Fig 2**).

### Relative quantification of expression of FMR1 RNA transcripts in granulosa cells among 5 isoform primer sets

**Fig 3:**
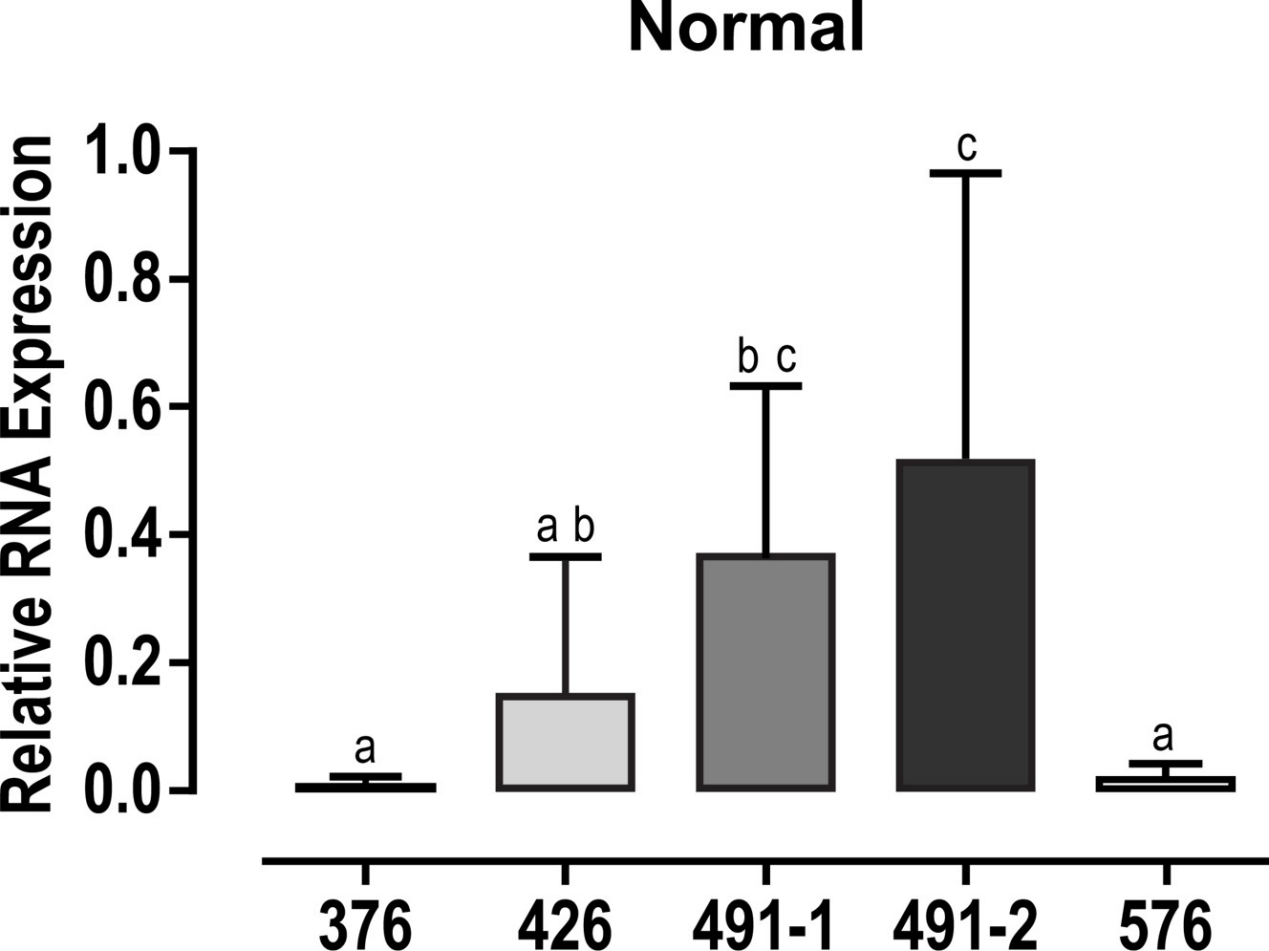
Relative expression of FMR1 gene. FMR1 RNA expression using different set of primers in granulosa cells from women with normal alleles of FMR1. Different characters indicate significant statistical difference between columns.

In luteinized granulosa cells from women with *norm FMR1* allele (CGG_n = 26–34_) FMR1 expression of RNA transcripts was significantly different among the five primer sets (p<0.001). Tukey post hoc analysis demonstrated products using primer sets 491–2 (0.512 ± 0.45) and 491–1 (0.364 ± 0.28) had greater quantitative relative expression of RNA compared to primer sets 376 (0.008 ± 0.01; p = 0.002) and 576 (0.017 ± 0.03; p< 0.001). Products using primer set 426 (0.148 ± 0.22; p = 0.09) demonstrated a trend toward increased RNA expression compared to primers 376 and 576 but did not reach statistical significance. (Fig 3)

### Relative expression of isoforms of the FMR1 gene in granulosa cells

**Fig 4:**
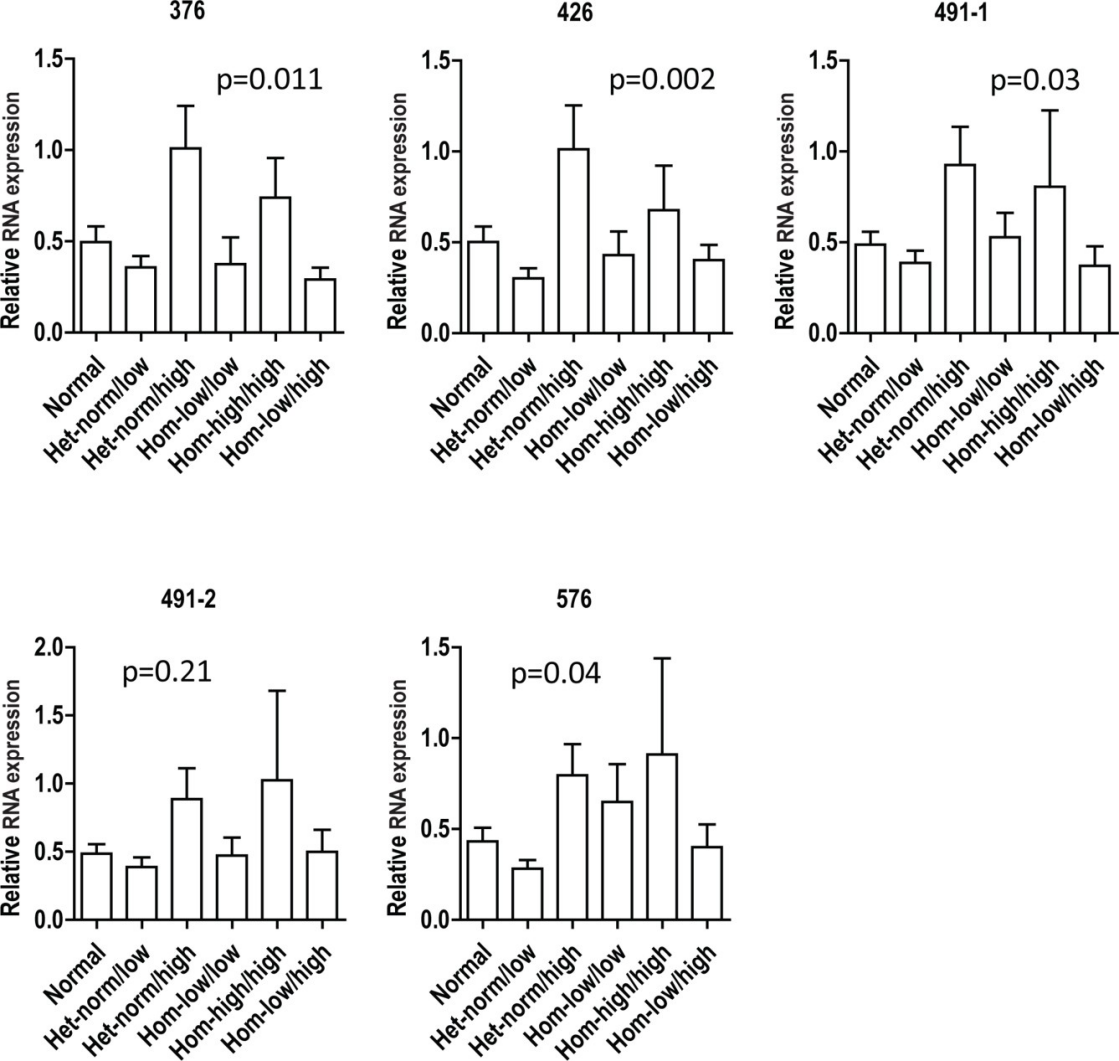
Relative expression of FMR1 genes: 6 sub-genotypes. FMR1 RNA expression using different set of primers in mural granulosa cells from women in 6 FMR1 sub-genotypes. Data were analyzed by 2-ΔΔCT method and normalized with 18S rRNA and then normalized across different PCRs to one patient as a control.

There was no significant difference in the distribution of expression of FMR1 RNA isoforms from mural granulosa cells between egg donors and patients or with infertility diagnosis or any racial grouping, although this data set may have been too small to detect such differences. Regression analysis of the lower FMR1 allele revealed a significant association of increased RNA expression among the combined isoforms with increasing number of FMR1 CGG repeats (p = 0.001). This association persisted when adjusting for age and for all potential confounders. A General Linear Model was used to adjust for repeated measures in individual patients did not reveal the same association (p = 0.07). The expression of FMR1 RNA isoforms from mural granulosa cells of various FMR1sub-genotypes, revealed that the 4 groups of FMR1 RNAs, containing a mixture of various isoforms, were differentially expressed in mural granulosa cells, except for the set of 491–2 which is one of the two predominant groups (Fig 4 and S1 Table).

### Relative expression of FMR1 genes: Low vs. Non-Low

The expression of FMR1 RNA isoforms from mural granulosa cells was reduced among women with an FMR1 allele that had fewer than 26 CGG repeats (“Low”) (p = 0.01). This finding was not changed by adjusting for age, race or type of infertility.

**Fig 5:**
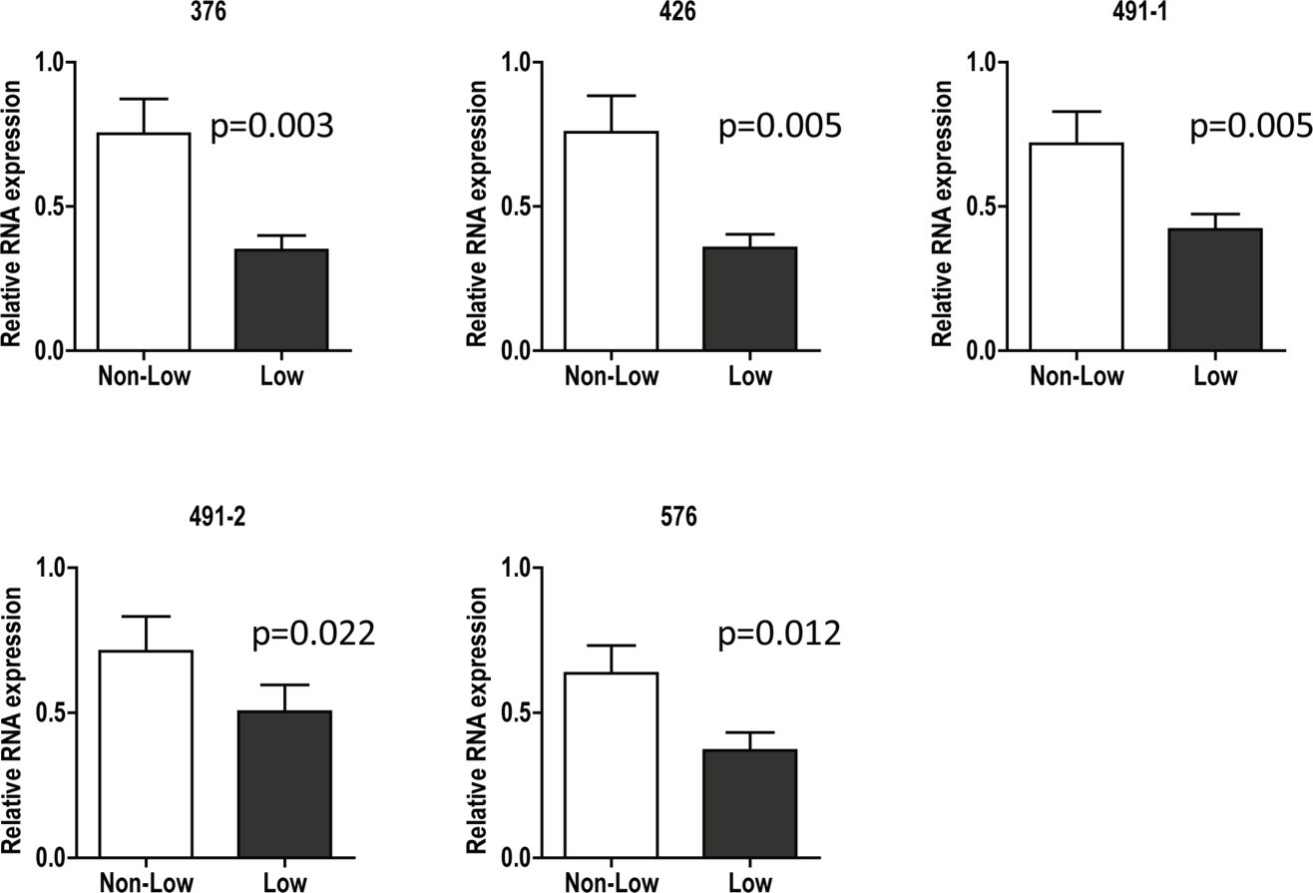
Relative expression of FMR1 genes: Low vs. Non-Low. FMR1 RNA expression using different set of primers in mural granulosa cells from women in low and non-low FMR1 groups. Data were analyzed by 2-ΔΔCT method and normalized with 18S rRNA and then normalized across different PCRs to one patient as a control.

Fig 5 illustrates that all isoforms of the *FMR1* RNA were significantly lower in women with *low* alleles (*low* sub-genotypes) than in women who carried no *low* alleles (see S2 Table).

## Discussion

We found differential quantitative expression of *FMR1* RNA isoforms in the granulosa cells of women undergoing oocyte retrieval following ovulation induction for IVF. Our main focus on women with *low FMR1* alleles was motivated by our previous observations that this genotype was associated with negative effects on female reproductive success [19–21, 30]. The observed clinical effect of FMR1 within the normal range remains controversial [23], though even those authors found that lower AFC, lower AMH levels and fewer oocytes were retrieved in the presence of fewer CGG repeats [23]. This study now reveals that in luteinized granulosa cells of women with *low FMR1* alleles quantitative expression of many RNA isoforms is significantly reduced in comparison to women who do not carry *low FMR1* alleles (*non-low* group).

When investigating associations between *FMR1* RNA isoforms and CGG_n_, the literature has previously focused on premutation (CGG_n~55–200_) and full mutation (CGG_n>200_) carriers. In male premutation carriers, a significant increase in *FMR1* RNA transcripts was observed in both peripheral blood mononuclear cells and brain tissue compared to normal controls for all groups of isoforms [31]. In contrast, for patients with full mutations the *FMR1* gene is hyper-methylated extending to the adjacent promoter region which leads to the silencing of its gene transcription [2, 4, 15, 32] and loss of FMRP.

Information about expression and changes of FMRP (and isoforms) in the literature in what is widely considered normal CGG_n<55_ range is sparse. By combining several sets of primers, we observed that this supposedly normal group of patients functionally can be further sub-divided according to recently described variants that have been associated with changes in “ovarian” function [19, 20, 33].

This study reveals that quantitative expression of *FMR1* gene RNA transcripts is decreased in constricted *low* alleles. Significantly lower expression at *low* CGG _n<26,_ and complete silencing at full mutation range (CGG_n>200_), suggests that expression may be affected at both extremes of CGG_n_. Yet, with expansion into the premutation range of CGG _n~55–200_ gene expression increases, suggesting that *FMR1* RNA expression may, after all, be more precisely regulated than has been so-far appreciated.

How such fine tuning is achieved in response to relatively minor changes in CGG_n_ remains to be determined. One can hypothesize that minor changes in CGG_n_ induce on/off switches for transcription of one or more isoforms, modulate the splicing site, and as a consequence alter expression or patterns of various isoforms. Altered RNA expression and the ratio of the corresponding isoforms may furthermore affect the overall functions of FMRP and regulate the expression of some steroidogenic enzymes and hormonal receptors in patients from each sub-genotype.

In this study we examined the relative expression of isoforms using 5 sets of primers. Due to sequence homology, each primer may amplify multiple isoforms and, therefore, each PCR product was potentially composed of multiple isoforms. Each group of *FMR1* isoforms which shared the same protein sequence at specific regions was obviously different, with two groups predominant and others relatively less abundant.

It is known that FMRP acts on various gene transcriptions mainly as a RNA-binding protein suppressor. Exploring the potential targets of FMRP in ovarian cells will offer further insights into *FMR1* gene effects on functional ovarian reserve, the ovarian aging process, female fertility, IVF outcomes and, potentially, also autoimmunity since all of these clinical phenotypes have been associated with specific *FMR1* genotypes [19–21, 30].

However, it is possible that the FMR1 RNA is not only acting as a functional messenger to produce FMRP but is also functioning in the regulation of genome organization and gene expression.[34] Non-coding RNA transcribed from protein coding loci may effect epigenetic processes without translation to a protein product. Thus, significant variations in FMR1 RNA transcription and expression may have biological significance even in the absence of knowledge of associated effects on FMRP.

Among all six genotypes, *hom* genotypes are uncommon and sub-genotypes even more so [*hom* sub-genotypes *high/high*, *high/low* and *low/low* are only found in less than 10% of women [35]]. Due to the small sample sizes of the *hom* groups, especially *hom-high/high* group, the expression of all isoforms examined in these groups showed large variation. Though we still observed significant differences for majority of the genes examined here, the conclusion of various isoforms in all “ovarian” *FMR1* genotypes can only be supported with much larger patient populations. We, therefore, are continuing the recruitment of more patients.

## Conclusions

In conclusion, we demonstrate evidence that within what currently is considered a normal range for CGG_n_ women diverge in transcription of *FMR1* RNA in accordance with recently described “ovarian” variants of the gene [19–21, 33, 35]. Moreover, this study convincingly demonstrates that within this “ovarian” mutation classification, *low FMR1* alleles demonstrate clearly lower expression than *non-low* alleles. Changes in RNA level and/or ratio of various isoforms of the *FMR1* gene may regulate, either through epigenetic processes or via the translation and cellular localization of FMRP the expression of steroidogenic enzymes and hormonal receptors, leading to ovarian dysfunction and possible infertility. Further investigation of these changes should lead to an improved understanding of contributions of the *FMR1* gene to physiologic and premature ovarian aging and female infertility.

## Supporting information

S1 Table Relative expression of FMR1 genes: 6 sub-genotypes. FMR1 RNA expression using different set of primers in mural granulosa cells from women in 6 FMR1 sub-genotypes. Data were analyzed by 2^-ΔΔCT^ method and normalized with 18S rRNA and then normalized across different PCRs to one patient as a control. Shaded cells are significantly difference in means in the Tukey's post hoc HSD test.
(DOCX)

S2 Table Relative expression of FMR1 genes: Low vs. Non-Low. FMR1 RNA expression using different set of primers in mural granulosa cells from women in low and non-low FMR1 groups. Data were analyzed by 2^-ΔΔCT^ method and normalized with 18S rRNA and then normalized across different PCRs to one patient as a control. All isoforms of the FMR1 RNA were significantly lower in women with low alleles (low sub-genotypes) than in women who carried no low alleles. Significance adjusted for age.
(DOCX)

S1 Data FMR1 isoform Data. Data for FMR1 isoforms including: ID, Age, Race, Donor/ Patient, FMR1 low/ non-low, FMR1 sub genotype, Low Allele repeat number, High Allele repeat number, Age at infertility diagnosis, Infertility diagnosis, FMR1 isoforms: 376, 426, 491–1, 491–2, 576.
(XLSX)
