# Supplementary material for: Reduced RNA expression of the FMR1 gene in women with low (CGGn<26) repeats
Source: PLoS One. 2018 Dec 21;13(12):e0209309. doi: 10.1371/journal.pone.0209309 (PMC6303073; doi:10.1371/journal.pone.0209309)
Supplement: S1 Table — FMR1 RNA expression using different set of primers in mural granulosa cells from women in 6 FMR1 sub-genotypes. Data were analyzed by 2-ΔΔCT method and normalized with 18S rRNA and then normalized across different PCRs to one patient as a control. Shaded cells are significantly difference in means in the Tukey's post hoc HSD test. (DOCX) [file pone.0209309.s001.docx]

|  | Normal | Het/High | Het/Low | Hom/High | Hom/Low | Hom/Low-High | P-value | P-value Age Adjusted |
| --- | --- | --- | --- | --- | --- | --- | --- | --- |
| N | 25 | 23 | 25 | 3 | 11 | 11 |  |  |
| 376 | 0.6 ± 0.4 | 1.0 ± 1.1 | 0.3 ± 0.4 | 0.7 ± 0.4 | 0.4 ± 0.4 | 0.4 ± 0.3 | 0.016 | 0.011 |
| 426 | 0.6 ± 0.5 | 1.0 ± 1.2 | 0.3 ± 0.4 | 0.7 ± 0.4 | 0.4 ± 0.4 | 0.5 ± 0.3 | 0.028 | 0.021 |
| 491-1 | 0.7 ± 0.5 | 1.1 ± 1.3 | 0.4 ± 0.5 | 0.8 ± 0.7 | 0.5 ± 0.4 | 0.5 ± 0.4 | 0.042 | 0.032 |
| 491-2 | 0.8 ± 0.7 | 1.0 ± 1.2 | 0.4 ± 0.5 | 1.0 ± 1.1 | 0.7 ± 0.8 | 0.6 ± 0.4 | 0.239 | 0.213 |
| 576 | 0.6 ± 0.5 | 1.0 ± 1.1 | 0.3 ± 0.5 | 0.9 ± 0.9 | 0.6 ± 0.6 | 0.5 ± 0.3 | 0.050 | 0.040 |
|  | *Shaded cells are significantly difference in means in the Tukey's post hoc HSD test | | | | | | | |

S1 Table 1
