# Supplementary material for: Reduced RNA expression of the FMR1 gene in women with low (CGGn<26) repeats
Source: PLoS One. 2018 Dec 21;13(12):e0209309. doi: 10.1371/journal.pone.0209309 (PMC6303073; doi:10.1371/journal.pone.0209309)
Supplement: S2 Table — FMR1 RNA expression using different set of primers in mural granulosa cells from women in low and non-low FMR1 groups. Data were analyzed by 2-ΔΔCT method and normalized with 18S rRNA and then normalized across different PCRs to one patient as a control. All isoforms of the FMR1 RNA were significantly lower in women with low alleles (low sub-genotypes) than in women who carried no low alleles. Significance adjusted for age. (DOCX) [file pone.0209309.s002.docx]

S2 Table 2

|  | Low | Non-Low | P-value | P-Value  Age Adjusted |
| --- | --- | --- | --- | --- |
| N | 47 | 51 |  |  |
| 376 | 0.4 ± 0.4 | 0.7 ± 0.8 | 0.003 | 0.003 |
| 426 | 0.4 ± 0.4 | 0.8 ± 0.9 | 0.005 | 0.005 |
| 491-1 | 0.5 ± 0.4 | 0.9 ± 0.9 | 0.006 | 0.005 |
| 491-2 | 0.5 ± 0.6 | 0.9 ± 1.0 | 0.024 | 0.022 |
| 576 | 0.4 ± 0.5 | 0.8 ± 0.8 | 0.013 | 0.012 |
